# Transactions of the Medical Society of the State of Pennsylvania, at Its Annual Session, Held in the City of Philadelphia, May, 1852

**Published:** 1852-12

**Authors:** 


*Transactions of the Medical Society of the State of Pennsylvania, at its Annual Session, held in the city of Philadelphia, May,* 1852. *Volume Second.*

The Transactions of the Medical Society of the State of Pennsylvania, for the year 1852, make a volume of 146 pages. It contains the address of the late president, Dr. Charles Innes, and reports from the various counties of the state. The reports embody considerable interesting and useful information respecting the topography of different sections of the state, and diseases that have prevailed during the preceding year.

Dr. Hiram Carson, of Montgomery Co., was elected president for the present year.

The society is evidently in a flourishing condition.

